# Failure of oral anti-Xa to prevent non-bacterial thrombotic endocarditis in cancer: case report and literature review

**DOI:** 10.1093/ehjcr/ytaf463

**Published:** 2025-09-18

**Authors:** Mariam Benjelloun, Edith Jottrand, Emmanuel Joly, Attilio Leone, Philippe Van De Borne

**Affiliations:** Cardiology Department, Erasme Hospital, Route de Lennik 808, Brussels 1070, Belgium; Cardiology Department, CHU Tivoli, Av. Max Buset 34, 7100 La Louvière, Belgium; Cardiology Department, CHU Tivoli, Av. Max Buset 34, 7100 La Louvière, Belgium; Cardiology Department, CHU Tivoli, Av. Max Buset 34, 7100 La Louvière, Belgium; Cardiology Department, CHU Tivoli, Av. Max Buset 34, 7100 La Louvière, Belgium

**Keywords:** Marantic endocarditis, Pulmonary adenocarcinoma, Pembrolizumab, Stroke, Hypercoagulability, Case report

## Abstract

**Background:**

Marantic endocarditis (ME) is a rare but potentially fatal complication of hypercoagulable states, posing a significant diagnostic challenge in oncology. Timely detection and appropriate treatment are essential to prevent complications and improve clinical outcomes. In neoplastic contexts, tumour cells can activate the coagulation cascade, leading to thromboembolic events that serve as critical warning signs. A multidisciplinary approach is vital for effective management of this condition.

**Case summary:**

A 64-year-old woman, recently diagnosed with non-small cell lung carcinoma, was receiving anticoagulation therapy with Rivaroxaban for right iliac-femoral deep vein thrombosis. She developed progressive symptoms, including visual disturbances and dyspnoea. Magnetic resonance imaging of the brain showed ischaemic lesions in the territory of the right posterior cerebral artery and a pulmonary-CT angiogram identified severe bilateral pulmonary embolism. A transoesophageal echocardiography revealed vegetation on the mitral valve, consistent with ME. Switching from Rivaroxaban to low molecular weight heparin temporarily stabilized her hypercoagulable state, but the rapid progression of her underlying malignancy led to her death within 5 months of the diagnosis.

**Discussion:**

This rare case of ME in a cancer patient, despite treatment with a direct oral anticoagulant, raises important considerations. Although cases are scarce in the literature, it highlights the complexities of anticoagulation management in oncology, suggesting that the hypercoagulable state may surpass the protective effects of these medications. This case underscores the need for enhanced cardiac monitoring and a tailored anticoagulation approach, even for patients under anticoagulant therapy. It advocates for re-evaluating follow-up protocols and anticoagulant selection in this clinical context.

Learning points

1. Stroke, thromboembolic events, and hypercoagulability in cancer patients, especially those with adenocarcinoma, serve as an alarming signal, necessitating prompt investigation for marantic endocarditis to prevent life-threatening complications.
2. The inability of oral anti-Xa anticoagulants to avert non-bacterial thrombotic endocarditis (NBTE) in cancer patients underscores the urgent need for vigilant evaluation and alternative treatment approaches. The absence of clear guidelines for marantic endocarditis management underscores the urgent need for standardized protocols to enhance patient outcomes.

## Introduction

Non-bacterial thrombotic endocarditis (NBTE) is a rare and often underdiagnosed condition characterized by sterile valvular vegetations, typically associated with malignancy and systemic embolic events. We report the case of a 64-year-old woman with metastatic lung adenocarcinoma who developed NBTE while on rivaroxaban, later complicated by pulmonary embolism and ischaemic stroke. Mitral valve vegetations were identified on imaging and the diagnosis was established based on clinical, imaging, and laboratory findings. Despite a switch to low molecular weight heparin, her condition deteriorated. This case highlights the diagnostic and therapeutic challenges of NBTE in oncology and the need for individualized anticoagulation strategies.

## Summary figure

**Table:** 

Date	Events
**27/01/2023**	Right femoral vein thrombosis. Initiation of Rivaroxaban (15 mg twice daily for 21 days, followed by 20 mg once daily).
**31/01/2023**	Chest angioscanner: Discovery of a spiculated pulmonary nodule in the left lower lobe measuring 23×16 mm with mediastinal and hilar lymphadenopathy.
**03/02/2023**	Positron Emission Tomography—Computed Tomography (PET-CT) : Revealing the pulmonary lesion with mediastinal and hilar lymphadenopathy. No metastases detected.
**10/02/2023**	Endobronchial ultrasound (EBUS): Non-small cell carcinoma (NSCLC - TTF1 -; P40 -; PD-L1 + > 50%; EGFR -). No mutations detected in the EGFR gene. No mutations were detected in codon V600 of the BRAF gene.
**20/02/2023**	Cardio-oncology consultation: Transthoracic echocardiography: Preserved left ventricular ejection fraction (LVEF)—Simpson Bp 60%; no significant valvular disease.
**10/03/2023**	Pembrolizumab (first cycle)
**29/03/2023**	Emergency visit: Visual disturbances + dyspnoea.
**30/03/2023**	Chest CT angiogram: Severe bilateral pulmonary embolism at the segmental level. Stop Rivaroxaban and start low molecular weight heparin.
**05/04/2023**	Brain MRI: Ischaemic stroke in the territory of the right posterior cerebral artery.
**13/04/2023**	Transthoracic echocardiogram (TTE): Discovery of a mass extending over the anterior mitral leaflet, measuring 18×7 mm and moderate mitral regurgitation. PISA: Surface orifice regurgitation (SOR) 0.23 cm^2^, Volume of regurgitation (VOR) 39 mL.
**14/04/2023**	Transoesophageal echocardiogram (TEE): Homogeneous mass extending on the anterior mitral leaflet measuring 21×7 mm associated with moderate mitral regurgitation (SOR 0.25 cm^2^, VOR 34 mL).
**14/04/2023**	Heart Team: Diagnosis established: marantic endocarditis in the context of pulmonary neoplasia.
**14/06/2023**	Chest angioscanner reveals an increase in the left hilar mass, enlargement of the pulmonary nodule, and the presence of multiple embolic images in the third-order branches of the pulmonary arteries bilaterally.
**16/06/2023**	Follow-up TTE: Further enlargement of the mass extending on the anterior mitral leaflet measuring 18×15 mm + new mass measuring 8.5×4.0 mm on the posterior mitral leaflet. Increased mitral insufficiency.
**07/2023**	Patient's Death At Home Under Palliative Care.

## Case presentation

A 64-year-old woman arrived at the emergency department in March 2023, with visual disturbances and increasing dyspnoea (NYHA grade III). She had a history of non-small-cell lung-carcinoma diagnosed two months prior, treated with Pembrolizumab (first cycle in March 2023), and a right femoral vein thrombosis (3 months earlier) for which she was on Rivaroxaban (15 mg twice daily for 21 days, followed by 20 mg once daily). Cardiac auscultation indicated a regular heart rhythm without murmurs, and clinical examination revealed cachexia with right lower limb paresis. Laboratory tests revealed thrombocytopenia (128 000/mm^3^), elevated D-dimers (35200 ng/mL), and decreased fibrinogen (0,85 g/L) with no signs of inflammation (CRP—C-reactive protein 6,8 mg/L) or organ dysfunction.

A contrast-enhanced brain CT scan was normal, while a pulmonary CT angiogram revealed bilateral pulmonary embolism despite adequate anticoagulation. The patient was admitted to the neurology department for stroke assessment and began treatment with low molecular weight heparin (LMWH). A brain MRI confirmed an ischaemic stroke in the territory of the right posterior cerebral artery and excluded cerebral metastasis.

**Figure 1:**
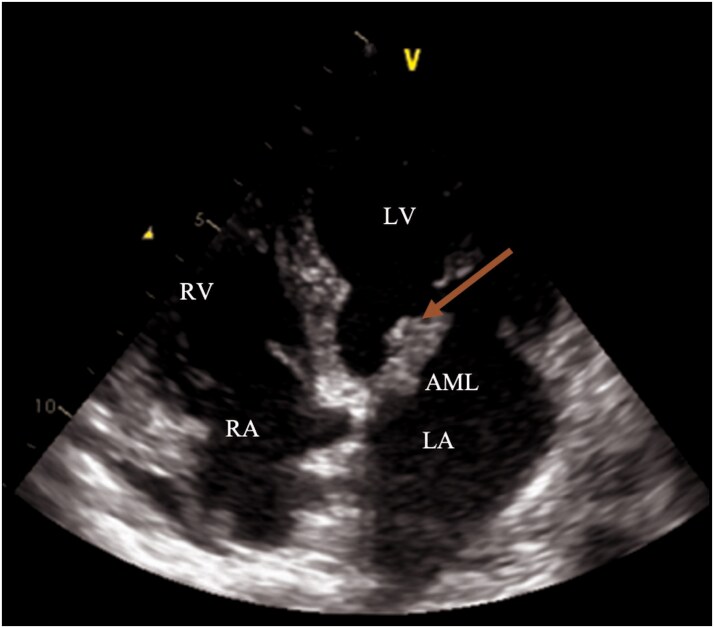
Transthoracic echocardiography (13 April 2023), four-chamber view. A mass extending on the anterior mitral leaflet is visualized, measuring 18× 7 mm (arrow). Anatomical landmarks: AML, anterior mitral leaflet; LA, left atrium; LV, left ventricle; RV, right ventricle; RA, right atrium.

In this context, a cardiovascular evaluation was requested to investigate a potential embolic source. A transthoracic echocardiogram performed revealed a mass attached to the anterior mitral leaflet (not present in the last echocardiogram conducted three weeks earlier). The mass was located on the ventricular side of the anterior mitral leaflet, measuring 18×7 mm, and was associated with moderate mitral regurgitation (*Figure 1*). Left ventricular function was preserved and no other masses were identified on the aortic or tricuspid valves.

**Figure 2:**
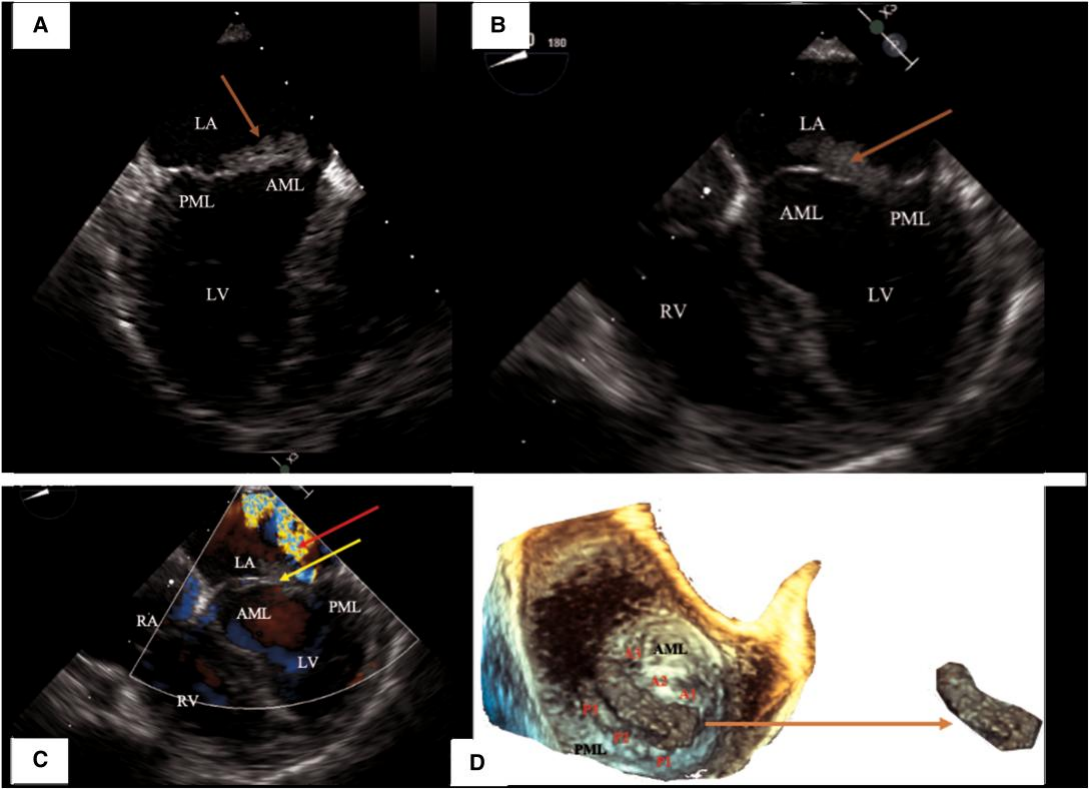
Transoesophageal echocardiography (TEE) (14 April 2023). (*A*) TEE zoom view (93°): mass measured 21× 7 mm (orange arrow). (*B*) TEE view (20°): mass measured at 21× 7 mm (orange arrow). (*C*) TEE view (20°): mitral regurgitation quantified as moderate by PISA method: SOR (Surface orifice regurgitation) 0.25 cm^2^, VOR (Volume of regurgitation) 34 mL, red arrow. Mass attached to AML (arrow). (*D*) TEE anterior view of the mitral valve—3D reconstruction (120°): mass attached to the anterior mitral leaflet (arrow). Anatomical landmarks: AML, anterior mitral leaflet (A1–A3); PML, posterior mitral leaflet (P1–P3); LA, left atrium; LV, left ventricle; RV, right ventricle; RA, right atrium.

Therefore, a transoesophageal echocardiogram (*Figure 2*) found a mobile vegetation measuring 21×7 mm, causing moderate eccentric mitral insufficiency along the lateral edge of the left atrium. Given the context of massive bilateral pulmonary embolism, the possibility of a patent foramen oval causing a paradoxical stroke was considered but the bubble test could not be conducted appropriately due to the patient's lack of cooperation and time constraints.

The differential diagnosis initially focused on infectious endocarditis; however, negative blood cultures and absence of inflammation made this unlikely. The diagnosis shifted towards marantic endocarditis (ME) or Libman–Sacks endocarditis, suggested by positive ANCA (1/320), hypothesizing that it was induced by immunotherapy. The search for antiphospholipid antibodies revealed a positive lupus anticoagulant, while anti-B2GP1 and anticardiolipin antibodies were negative. Considering the patient's history and the evolving neoplastic context, the hypothesis of ME was retained, and anticoagulation therapy with LMWH continued.

**Figure 3:**
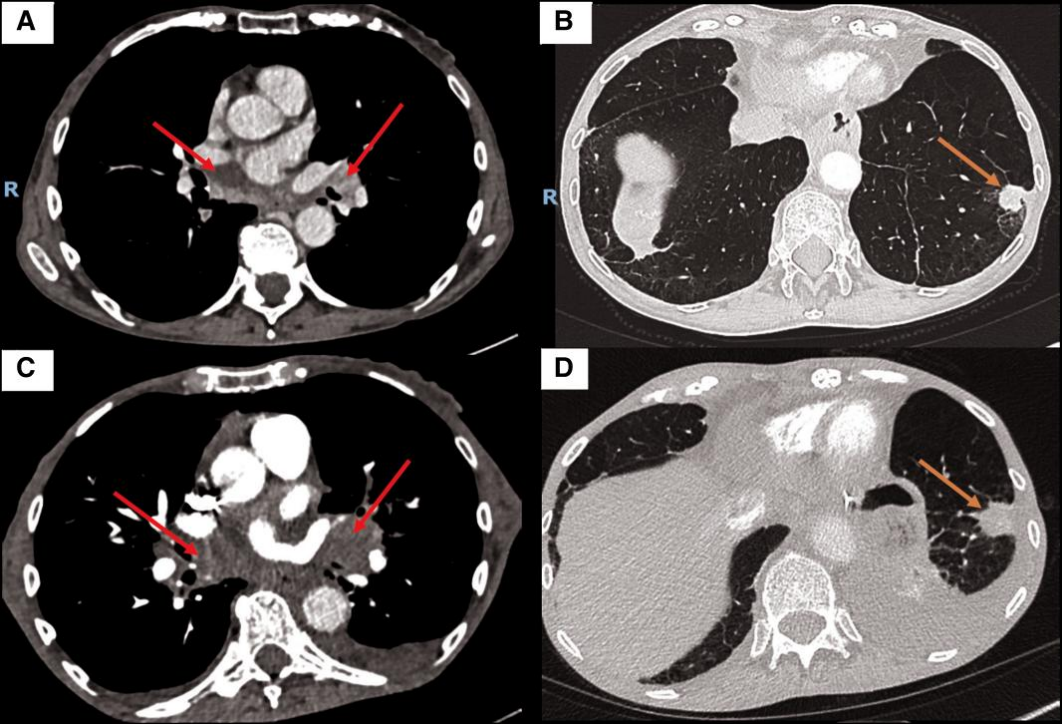
Chest angioscanner scans in lung (*B–D*) and mediastinal windows (*A–C*) from 31 January 2023 to 14 June 2023, showing increased size of the spiculated lesion in the left lower lobe (orange arrow) and progressive mediastinal lymphadenopathy (red arrow).

**Figure 4:**
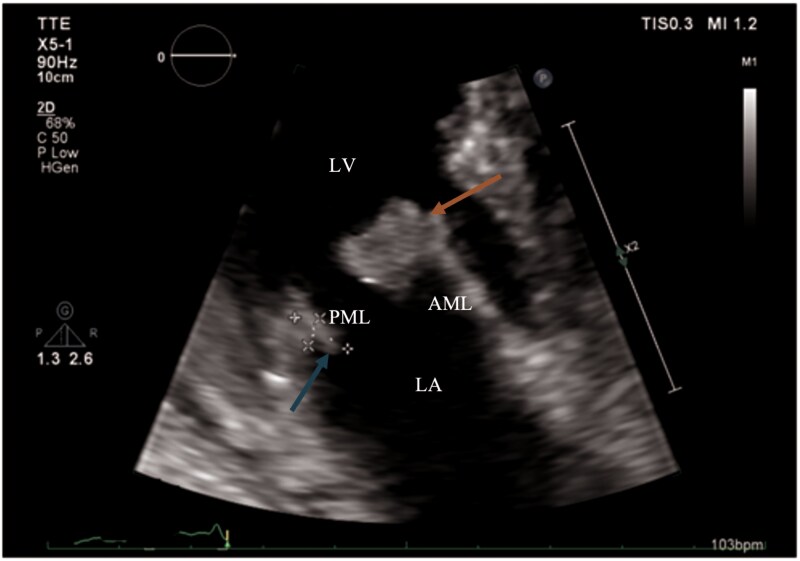
Transthoracic echocardiography (16 June 2023), three-chamber zoom view: mass on the anterior mitral leaflet (18× 15 mm, arrow) and new mass on the posterior mitral leaflet (8.5× 4.0 mm, arrow). Anatomical landmarks: AML, anterior mitral leaflet; PML, posterior mitral leaflet; LA, left atrium; LV, left ventricle.

The patient's management was complicated by her refusal of further examinations and chemotherapy, leading to cancer progression (*Figure 3*). Despite adequate heparin therapy, echocardiography revealed increasing mass on the anterior mitral leaflet and a new mass on the posterior mitral leaflet (*Figure 4*). Following psychiatric consultation, she expressed a wish to return home, where she passed away shortly afterward.

## Discussion

Non-bacterial thrombotic endocarditis (NBTE) features sterile thrombotic vegetations without microorganisms, inflammation, or valvular damage.^1^ ME is a rare complication in malignancies, particularly lung adenocarcinomas followed by gastric and pancreatic adenocarcinomas, and then breast and genitourinary cancer. A multi-factorial hypercoagulable state often underlies NBTE and may present with signs of disseminated intra-vascular coagulation, as in our patient.

Given systemic emboli and mitral vegetations, infective endocarditis was first suspected. However, absence of fever, inflammatory markers, and persistently negative blood cultures made this unlikely, aligning with ESC guidelines.^2^ Positive ANCA and lupus anticoagulant raised suspicion for Libman–Sacks endocarditis, typically associated with systemic lupus or antiphospholipid syndrome. These immune-mediated lesions preferentially affect the mitral valve and are best visualized on transoesophageal echocardiography. Our patient had no autoimmune history but was receiving pembrolizumab, an ICI known to trigger *de novo* autoimmunity.^3^ The 2022 ESC guidelines list ICI-related myocarditis and vasculitis among cardiovascular risks (Lyon *et al*., 2022). Still, no established link exists between ICIs and Libman–Sacks. A possible false-positive lupus anticoagulant result due to rivaroxaban, and absent systemic features, made this diagnosis unlikely. NBTE is the most plausible diagnosis in malignancy patients with systemic emboli, sterile vegetations, and negative cultures.

Initially prescribed for DVT (deep vein thrombosis), rivaroxaban was continued after cancer diagnosis due to clinical stability and patient preference. The Caravaggio trial supports DOACs in cancer-associated thrombosis.^4^ Following the pulmonary embolism, LMWH was initiated in accordance with the 2023 ESC guidelines, which recommend LMWH or UFH for the management of NBTE and advice against the use of DOACs.^2^ Anti-Xa levels were not measured, but dosing and compliance were verified.

A literature review identified only 10 ME cases under anticoagulation (*Table 1*): mean age 61.2, equal gender distribution, 80% on DOACs, 100% mitral valve involvement, 70% presented with stroke. This highlights both the rarity of ME under anticoagulants and the uncertainty surrounding optimal management.

**Table 1:** Cases of marantic endocarditis occurring despite prior oral anticoagulation in a neoplastic context

Author (year)	Age/sex	Cancer/pathology	Prior treatment (indication)	Initial presentation	Affected valve	Treatment	Outcome
F. Pons *et al.* (2010)^5^	Female/65 years	Lung adenocarcinoma (with supraclavicular lymphadenopathy) T1N2M + - no treatment	VKA (DVT)	Stroke	Mitral	UFH, then therapeutic cessation	Deceased
F. Pons *et al.* (2010)^6^	Male/63 years	Non-operable lung adenocarcinoma (T1N2M+) treated with radio chemotherapy (cisplatin and Alimta)	VKA (DVT)	NSTEMI, Stroke	Mitral + Aortic	LMWH	Deceased
F. Mantovani *et al.* (2017)^7^	Female/65 years	Pancreatic adenocarcinoma	Rivaroxaban (DVT and PE)	Recurrent PE	Mitral + Aortic	UFH then LMWH	Survival
Yoshiharu Soga *et al.* (2018)^8^	Male/69 years	Tubular adenocarcinoma of the stomach	Apixaban (DVT)	Stroke	Mitral	UFH then LMWH	Survival
Marissa *et al.* (2019)^6^	Female/63 years	Biliary adenocarcinoma	Rivaroxaban (DVT)	Stroke	Mitral + Aortic	Apixaban, then UFH (du to multiple emboli) and then LMWH	Survival
Sihan *et al.* (2020)^9^	Male/48 years	antiphospholipid syndrome (APS)	Apixaban (previous PE and DVT after failure of Warfarin and Rivaroxaban)	Stroke	Mitral	UFH + surgical (mitral valve replacement)	Deceased
Tosha Hedin *et al.* (2023)^10^	Male/54 years	Oesophageal adenocarcinoma	Rivaroxaban (AF)	–	Mitral	LMWH	Survival
Amit K. Mandal *et al.* (2023)^11^	Male/37 years	Antiphospholipid syndrome (APS)	Rivaroxaban (APS—initially on Warfarin)	Heart failure	Mitral + Aortic	VKA	Survival
Elias Akiki *et al.* (2023)^12^	Female/70 years	Stage IV lung adenocarcinoma (pleural metastasis, pericardial, mediastinal lymphadenopathy) Treatment: pembrolizumab and pemetrexed (chemotherapy)	Apixaban (AF and PE)	Stroke	Mitral	LMWH	Survival
William R Rankin *et al.* (2024)^13^	Female/78 years	Acute myeloid leukaemia	Apixaban (DVT)	Stroke	Mitral + Aortic	LMWH	Survival

DVT, deep vein thrombosis; NSTEMI, non-ST-elevation myocardial infarction; MR, mitral regurgitation; AI, aortic regurgitation; PE, pulmonary embolism; APS, antiphospholipid syndrome; LMWH, low molecular weight heparin; UFH, unfractionated heparin.

ME treatment centres on anticoagulation and cancer control. While DOACs like edoxaban and rivaroxaban inhibit factor Xa, they may be ineffective in this context. LMWH remains preferred in NBTE, as per ESC, ASCO, and Chest guidelines.^14^ The case reported by Akiki *et al*.^12^ supports this approach: NBTE under apixaban resolved after switching to enoxaparin. Venepally *et al*.^1^ found that progressive cancer was the strongest predictor of mortality, even with appropriate anticoagulation.

Prognosis depends on aetiology and extent. Our patient had advanced lung adenocarcinoma and mediastinal lymphadenopathy, associated with poor survival. Compared with previously reported cases, this presentation appears unusual, as it involved NBTE under rivaroxaban in a patient treated exclusively with pembrolizumab. Unlike Akiki *et al*., our patient worsened post-LMWH switch and died. This supports previous findings that associate poor outcomes with advanced or progressive disease.^1^

To our knowledge, few reports have considered Libman–Sacks endocarditis in ICI-treated patients without autoimmune disease, making this case noteworthy. This serological profile, possibly influenced by immunotherapy, illustrates a diagnostic complexity not previously addressed.

Though thrombotic events pre-dated pembrolizumab, PD-1 inhibitors may enhance thrombosis risk via endothelial dysfunction and immune activation.^15^ This supports close thrombotic monitoring in oncology patients receiving ICIs.

Given cancer progression and limited response to LMWH, antiplatelet therapy was considered. However, due to bleeding risk and the absence of guideline recommendations, it was not pursued. None of the reviewed cases reported using this strategy.

In conclusion, ME should be suspected in cancer patients with systemic embolism, sterile vegetations, and no infection signs. A comprehensive differential, guideline-based anticoagulation, and multimodal imaging are key. This case underscores the diagnostic and therapeutic uncertainty surrounding NBTE, reinforcing the need for evidence-based recommendations.

## Data Availability

All relevant data are included in the article.

## Lead author biography

My name is Mariam Benjelloun, and I am a young cardiology resident at Université Libre de Bruxelles, originally from Morocco. I am deeply passionate about electrophysiology, echocardiography, and valvular diseases. My commitment to these areas drives my desire to enhance my expertise and make meaningful contributions to the field of cardiology.
